# Diversity and population structure of a dominant deciduous tree based on morphological and genetic data

**DOI:** 10.1093/aobpla/plv103

**Published:** 2015-08-26

**Authors:** Qin-di Zhang, Rui-Zhi Jia, Chao Meng, Chao-Wen Ti, Yi-Ling Wang

**Affiliations:** College of Life Sciences, Shanxi Normal University, Linfen 041000, China

**Keywords:** *Acer grosseri* Pax., genetic diversity, genetic structure, morphological traits, sequence-related amplified polymorphism (SRAP)

## Abstract

Knowledge of the genetic diversity and structure of tree species across their geographic ranges is essential for sustainable use and management of forest ecosystems. *Acer grosseri* Pax., an economically and ecologically important maple species, is mainly distributed in North China. In this study, the genetic diversity and population differentiation of 24 natural populations of this species were evaluated using sequence-related amplified polymorphism markers and morphological characters. The results show that highly significant differences occurred in 32 morphological traits. The coefficient of variation of 34 characters was 18.19 %. Principal component analysis indicated that 18 of 34 traits explained 60.20 % of the total variance. The phenotypic differentiation coefficient (*V*_ST_) was 36.06 % for all morphological traits. The Shannon–Wiener index of 34 morphological characters was 6.09, while at the population level, it was 1.77. The percentage of polymorphic bands of all studied *A. grosseri* populations was 82.14 %. Nei's gene diversity (*H*_e_) and Shannon's information index (*I*) were 0.35 and 0.50, respectively. Less genetic differentiation was detected among the natural populations (*G*_ST_ = 0.20, *Φ*_ST_ = 0.10). Twenty-four populations of *A. grosseri* formed two main clusters, which is consistent with morphological cluster analysis. Principal coordinates analysis and STRUCTURE analysis supported the UPGMA-cluster dendrogram. There was no significant correlation between genetic and geographical distances among populations. Both molecular and morphological data suggested that *A. grosseri* is rich in genetic diversity. The high level of genetic variation within populations could be affected by the biological characters, mating system and lifespan of *A. grosseri*, whereas the lower genetic diversity among populations could be caused by effective gene exchange, selective pressure from environmental heterogeneity and the species' geographical range.

## Abstract (precis)

*Acer grosseri*, an economically and ecologically important maple species, is mainly distributed in North China. Based on Sequence Related Amplified Polymorphism (SRAP) markers and morphological characters, *A. grosseri* shows rich genetic diversity. Less genetic differentiation occurred between than within natural populations. All studied populations of *A. grosseri* formed two main clusters. There was no significant correlation between genetic and geographical distances among populations. The biological characters, mating system, gene exchange, and selective pressure from environmental heterogeneity are the main factors affecting the genetic population structure of *A. grosseri*.

## Introduction

Genetic diversity, adaptation and evolution of a species (Ma *et al*. 2008) can reflect the species' evolutionary history (e.g. shifts in distribution, habitat fragmentation and population isolation) and the interactions of various processes (such as mutation, genetic drift, mating system, gene flow and natural selection) (Slatkin 1987; Reed and Frankham 2003). Investigation of levels of genetic diversity in natural populations is an important precursor for the study of plant species (Stevens *et al*. 2007) and will provide insights into the evolution of the species (Ueno *et al*. 2000).

Estimates of genetic diversity and genetic variation can be indirectly carried out based on morphological information (Shehzad *et al*. 2009; Barro-Kondombo *et al*. 2010; Jugran *et al*. 2013); however, such estimates do not reliably reflect the real genetic variation because most morphological characters are greatly influenced by environmental factors, the developmental stage of the plant and largely unknown genetic control of polygenic inheritance morphological traits (Shehzad *et al*. 2009; Last *et al*. 2014). Despite this, morphological characterization is highly recommended as a first step before starting DNA-based studies (Hoogendijk and Williams 2001). Morphological methods are inexpensive and easily implemented, while molecular markers are more variable, less dependent on the environment and more informative at any developmental stage than morphological methods (Backes *et al*. 2003). Nowadays, evaluation of the genetic diversity and structure of plant populations is performed using both morphological characters and molecular markers (Feng *et al*. 2006; Barro-Kondombo *et al*. 2010; Hajmansoor *et al*. 2013; Wang *et al*. 2013).

*Acer grosseri* belongs to the Aceraceae and is an important ornamental perennial tree both economically and medicinally (Fang 1981; Liu 2000). It is mainly distributed in open forest between 1000 and 1600 m above sea level. This species has unisexual and dioecious flowers, key fruits and small nuts (Fang 1981; Liu 2000). *A. grosseri* can grow on different soil types, such as yellow brown soil, brown soil, brown forest soil, yellow cinnamon soil and cinnamon soil (Tian and Wang 2009). Being a main associate species (no-dominant species), the wild community of *A. grosseri* usually grows in the moist fertile acid soil of shady or semi-shady slopes, in the deciduous broad-leaved forests in the warm temperate zone of North China (Fang 1981). Due to environmental degradation and human destruction, the geographical distribution of *A. grosseri* is constantly decreasing. There is thus an urgent need to develop strategies for conservation and to effectively harness the potential of this species. In this context, knowledge about the genetic diversity and population structure of *A. grosseri* will be valuable (Joshi and Dhawan 2007; Jugran *et al*. 2013), and the conservation of particularly genetically rich populations will be useful (Jugran *et al*. 2013). However, previous studies have mainly focussed on the biological characteristics (Han *et al*. 2010), landscape use (Han *et al*. 2010), propagation (Jia *et al*. 2010), photosynthetic characteristics (Yuan and Yao 2012) and physiological traits (Gu *et al*. 2010) of *A. grosseri*.

We, therefore, conducted a genetic analysis of *A. grosseri* using sequence-related amplified polymorphism (SRAP) markers and morphological characters to (i) evaluate the morphological variability and diversity of 24 natural populations, (ii) investigate the genetic diversity and structure of *A. grosseri* populations and (iii) identify the factors that affect the current genetic diversity and differentiation of this natural genetic resource.

## Methods

### Plant sampling

**Figure 1.**
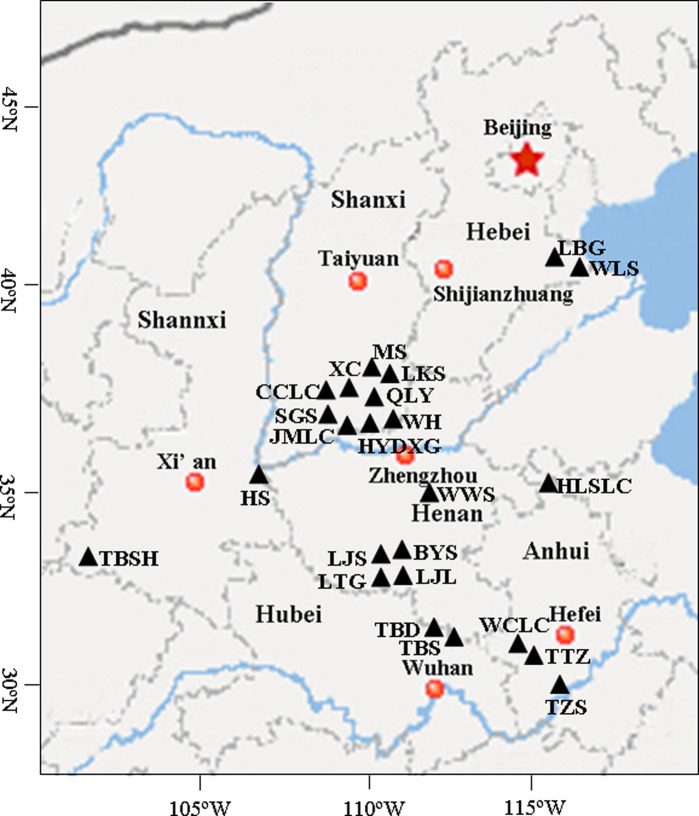
Map of sample sites of 24 *A. grosseri* populations. Location details are given in Table 3.

A total of 675 samples were collected from 24 natural populations of *A. grosseri*, covering almost its entire distribution range in North China (Fig. 1, Table 3). The individuals present at a location were treated as ‘a population’, a common approach in traditional genetic structure studies; thus, several locations were examined within the range. The sample sizes were determined based on different population sizes, ranging from 20 to 35 individuals (Table 3). Individuals within each population were separated from each other by at least 30 m. Leaves and fruits (seeds) were recorded separately for every sample site in numbered envelopes. Immediately after collection, labelled envelopes were placed in an ice-box and transported to the laboratory.

### Morphological analysis

Thirty-four morphological characteristics were determined, 19 traits from leaves, 11 from fruits and 4 from seeds. Quantitative measurements were carried out on 30 leaves and 30 fruits (seeds) per sample. Dimensions (length and width) for leaf, fruit and seed were measured by digital caliper with 0.1 cm precision. Small dimensions were measured by a digital Vernier calliper (Mitutoya, Japan) with 0.02 mm precision. Angles were measured using a protractor with 1° precision. Leaf area was measured using a leaf area metre with 0.01 cm^2^ precision. The morphological characters of all populations were measured in three replicates, and the mean value was used for the analysis. Thirty-four morphological variables were recorded as described in Table 1.

**Table 1.** The morphological traits measured among *A. grosseri* populations. LL, leaf length; LML, laminae length; LW, laminae width; LLW, laminae length/width; L^L^, length from leaf apex to the widest of leaf left; L_L_W, length from leaf apex to the widest of leaf left/leaf width; L_H_, height from leaf apex to the widest of leaf left; α, angle of leaf vein; *n*, leaf vein number; β, angle of leaf left; LAW, leaf apex width; LAH, leaf apex height; LAWH, leaf apex width/height; LAW_L_, leaf apex width/length from leaf apex to the widest of leaf left; LA, laminae area; LSL, leafstalk length; LSW, leafstalk width; LSLW, leafstalk length/width; LLL, laminae length/leafstalk length; KFSL, key fruit stalk length; FSL, fruit stalk length; KFL, key fruit length; KFW, key fruit width; KFLW, key fruit length/width; γ, angle of leaf base; AS, attachment scar; FL, fruit length; FW, fruit width; FT, fruit thickness; FLW, fruit length/width; SL, seed length; SW, seed width; ST, seed thickness; SLW, seed length/width; CV, coefficient of variation; *V*_ST_, differentiation coefficient of phenotypic traits; *H*_SW_, Shannon–Wiener index. * and **Indicates the value of the traits is significantly different (*F*-value ≥ *F*_0.05_ and *F*_0.01_, respectively).

Traits	Mean value	Standard deviation	*F*-value		CV (%)	*V*_ST_ (%)	*H*_SW_	PCA		
			Among populations	Within populations				PC1	PC2	PC3
LL	15.78	2.69	20.575**	3.121**	14.24	31.51	7.13	0.61	0.66	0.18
LML	10.80	1.89	31.629**	4.567**	14.30	44.84	7.13	0.72	0.58	0.26
LW	9.15	1.98	17.758**	2.544**	17.53	44.97	7.13	0.86	0.62	0.09
LLW	1.21	0.72	5.915**	2.833**	15.43	6.66	7.14	−0.33	−0.22	0.34
L^L^	5.44	1.27	10.081**	2.116**	17.95	37.62	7.13	−0.06	0.80	−0.10
L_L_W	0.62	0.55	18.675**	5.372**	15.20	8.12	7.12	−0.69	0.22	−0.14
L_H_	5.31	1.53	22.997**	3.880**	17.38	3.02	7.10	0.86	0.29	0.10
α	48.35	6.70	2.756**	0.962**	10.74	33.10	7.13	0.67	−0.14	−0.47
*n*	17.36	2.69	3.468**	1.120**	12.62	28.89	7.13	−0.55	0.26	0.25
β	49.24	6.41	5.154**	2.662**	9.51	23.32	7.13	0.02	0.38	−0.73
LAW	0.42	0.15	4.646**	2.647**	30.19	42.23	7.13	−0.08	0.45	−0.60
LAH	1.29	0.49	17.664**	1.646*	26.02	23.74	7.10	0.76	0.13	0.23
LAWH	0.37	0.22	11.199**	2.061**	37.83	44.43	7.09	−0.71	0.09	−0.22
LAW_L_	0.08	0.04	11.042**	4.709**	34.65	32.66	7.12	−0.55	−0.52	−0.02
LA	53.46	16.29	9.340**	3.222**	23.91	34.46	6.13	0.47	0.521	0.64
LSL	4.98	1.48	4.291**	1.409**	26.80	3.58	7.13	0.02	0.45	−0.12
LSW	1.42	0.30	2.895**	2.278**	18.81	37.95	7.13	0.46	0.67	−0.28
LSLW	3.62	1.36	4.070**	1.514**	30.76	48.58	7.12	−0.39	−0.14	0.11
LLL	2.34	0.81	4.322**	1.342**	29.48	28.09	6.12	0.63	0.02	0.32
KFSL	1.96	0.60	5.650**	0.873**	21.29	38.11	5.13	−0.37	0.28	0.10
FSL	0.64	0.27	16.799**	1.198**	22.38	64.63	5.09	−0.46	0.25	0.12
KFL	2.16	0.52	47.766**	1.356**	14.40	41.95	5.08	0.70	−0.22	−0.06
KFW	0.78	0.14	3.565**	3.134**	13.10	39.71	5.11	−0.39	0.33	−0.11
KFLW	2.81	0.68	18.684**	0.779	12.48	57.09	5.13	0.80	−0.32	−0.02
γ	115.35	15.96	8.631**	3.487**	9.68	44.64	6.10	−0.55	0.19	0.62
AS	3.95	0.41	1.404**	0.524**	6.84	26.89	5.13	0.27	−0.13	0.74
FL	8.03	1.32	2.698**	1.048	12.10	65.19	5.13	0.49	−0.07	0.50
FW	4.96	0.73	1.923*	1.370	10.91	30.13	5.13	−0.65	0.21	0.61
FT	2.18	0.70	9.473**	2.626**	24.55	32.79	5.12	0.56	−0.09	−0.45
FLW	1.65	0.32	1.989*	0.931	14.91	37.25	5.13	0.75	−0.23	−0.21
SL	6.01	1.19	3.400**	1.071**	14.51	55.74	4.13	0.64	−0.15	0.41
SW	3.03	0.63	20.388**	2.511**	12.64	42.06	4.12	0.83	−0.40	0.18
ST	1.88	0.59	16.478**	4.758**	13.07	52.12	4.12	0.44	0.01	−0.46
SLW	2.05	0.53	4.324**	2.310**	12.24	39.88	4.13	−0.70	0.49	0.07
Mean					18.19	36.06	6.09			
Eigen values								11.39	4.62	4.46
% of variance								33.49	13.60	13.11
Cumulative %								33.49	47.09	60.20

The morphological traits data were standardized. One-way analysis of variance (ANOVA) was performed using the Statistical Package for Social Sciences program (SPSS 17.0, SPSS Inc., USA). Coefficients of variation (CV %) were determined as indicators of morphological variability. The simple correlation coefficient was calculated to determine the relationships between the studied morphological variables using the Pearson correlation coefficient.

Factor analysis was based on principal components to provide a reduced dimension model indicating differences measured among groups. This analysis has been widely used as a data reduction method to analyse multiple data on morphological characteristics and it has proven to be a valuable tool to understand relationships between characteristics and between populations (Khadivi-Khub *et al*. 2012). Principal component analysis (PCA) allows the evaluation of multicollinear data and determination of the traits most suitable for classification (Iezzoni and Pritts 1991). Based on morphological traits of *A. grosseri*, PCA was investigated using SPSS software.

The phenotypic differentiation coefficient (*V*_ST_) was assessed using the formula $V_{\mathrm{ST}}=({\overset{´}{o}}_{t/s}^{2})/({\overset{´}{o}}_{t/s}^{2}+{\overset{´}{o}}_{t}^{2}),$ where ${\overset{´}{o}}_{t/s}^{2}$ is the percentage of variance between populations and ${\overset{´}{o}}_{t}^{2}$ is the percentage of variance within populations.

The phenotype diversity (Shannon–Wiener information index, *H*_sw_) of each morphological trait and each population was evaluated using BIO-Dap software.

The morphological similarity coefficients according to the Euclidean method were calculated using the SIMINT program of the numerical taxonomy multivariate analysis system NTSYS-pc v2.10 (Rohlf 2000), and the dendrograms were constructed with the SAHN clustering program using the unweighted pair-group method with arithmetic means (UPGMA).

### Molecular analysis

#### DNA extraction and SRAP amplification

Genomic DNA was extracted using a modified cetyl tri-methyl ammonium bromide method (Doyle and Doyle 1987), with 1 g polyvinyl pyrrolidone added to remove phenolics present in the sample. The DNA concentration was estimated using a Nanodrop (Thermo Fisher, USA). DNA quality was checked by gel electrophoresis on 0.8 % agarose gel. A working DNA concentration of 50 ng μL^−1^ was prepared and stored at 4 °C until use.

The SRAP analysis was carried out according to protocols previously established by Li and Quiros (2001), with minor modifications. Eighty-eight SRAP primer combinations (Invitrogen Biotech (Shanghai) Co., China) were initially screened in 24 representative samples from all studied populations. Primer combinations were excluded from the study if their banding patterns were difficult to score or if they failed to amplify consistently in all lines. Of the 88 SRAP primer pairs, 20 primer combinations that produced consistent amplifications and clear polymorphic bands were selected to amplify the rest of the samples (Table 2). All samples were amplified at least three times if the initial amplification failed.

**Table 2.** Sequences of SRAP forward and reverse primers used in the present study.

Primer combination	Primer sequence	Primer combination	Primer sequence
Me1/Em3	3′-TGAGTCCAAACCGGATA-5′	Me3/Em4	3′-TGAGTCCAAACCGGAAT-5′
	5′-GACTGCGTACGAATTGAC-3′		5′-GACTGCGTACGAATTTGA-3′
Me1/Em4	3′-TGAGTCCAAACCGGATA-5′	Me3/Em10	3′-TGAGTCCAAACCGGAAT-5′
	5′-GACTGCGTACGAATTTGA-3′		5′-GACTGCGTACGAATTCAG-3′
Me1/Em9	3′-TGAGTCCAAACCGGATA-5′	Me5/Em8	3′-TGAGTCCAAACCGGAAG-5′
	5′-GACTGCGTACGAATTCGA-3′		5′-GACTGCGTACGAATTCTG-3′
Me1/Em10	3′-TGAGTCCAAACCGGATA-5′	Me5/Em10	3′-TGAGTCCAAACCGGAAG-5′
	5′-GACTGCGTACGAATTCAG-3′		5′-GACTGCGTACGAATTCAG-3′
Me1/Em11	3′-TGAGTCCAAACCGGATA-5′	Me6/Em9	3′-TGAGTCCAAACCGGGCT-5′
	5′-GACTGCGTACGAATTCCA-3′		5′-GACTGCGTACGAATTCGA-3′
Me2/Em2	3′-TGAGTCCAAACCGGAGC-5′	Me6/Em8	3′-TGAGTCCAAACCGGGCT-5′
	5′-GACTGCGTACGAATTTGC-3′		5′-GACTGCGTACGAATTCTG-3′
Me2/Em3	3′-TGAGTCCAAACCGGAGC-5′	Me6/Em11	3′-TGAGTCCAAACCGGGCT-5′
	5′-GACTGCGTACGAATTGAC-3′		5′-GACTGCGTACGAATTCCA-3′
Me2/Em4	3′-TGAGTCCAAACCGGAGC-5′	Me7/Em3	3′-TGAGTCCAAACCGGTCC-5′
	5′-GACTGCGTACGAATTTGA-3′		5′-GACTGCGTACGAATTGAC-3′
Me2/Em6	3′-TGAGTCCAAACCGGAGC-5′	Me7/Em6	3′-TGAGTCCAAACCGGTCC-5′
	5′-GACTGCGTACGAATTGCA-3′		5′-GACTGCGTACGAATTGCA-3′
Me2/Em8	3′-TGAGTCCAAACCGGAGC-5′	Me8/Em8	3′-TGAGTCCAAACCGGTGC-5′
	5′-GACTGCGTACGAATTCTG-3′		5′-GACTGCGTACGAATTCTG-3′

All PCRs were performed in a PTC-200TM programmable Thermal Controller (Bio-Rad, USA). The SRAP reaction mixtures (total volume = 10 μL) contained 50 ng DNA, 2× MasterMix (0.2 mM dNTPs, 3 mM MgCl_2_, 1× PCR buffer and 0.1 U Taq DNA polymerase) and 0.6 mM of both forward and reverse primers. The PCR amplification programme was as follows: 5 min of pre-denaturation at 94 °C; 1 min of denaturation at 94 °C; five cycles of 1 min of denaturation at 94 °C, 1 min of annealing at 35 °C and 1 min of elongation at 72 °C; then 35 cycles of denaturation for 1 min at 94 °C, annealing for 1 min at 50 °C, elongation for 1 min at 72 °C and a final elongation step of 5 min at 72 °C. Sequence-related amplified polymorphism products were analysed on 3 % non-denatured polyacrylamide gels in 1× TBE buffer running at 120 V constant voltage for 1 h and then silver stained according to previously reported procedures (Liu *et al*. 2008; Cai *et al*. 2011).

#### Genetic data analysis

All clearly detectable SRAP product bands were scored as either presence (1) or absence (0), and the matrix of SRAPs data was assembled. Only reproducible and well-defined bands were scored.

Population genetic parameters were analysed using POPGENE v3.2 (Yeh *et al*. 1999) to determine the percentage of polymorphic bands (PPBs), observed number of alleles (*N*_a_), effective number of alleles (*N*_e_), Nei's gene diversity (*H*_e_), Shannon's information index (*I*) and genetic differentiation between populations (*G*_ST_). In addition, pair-wise *N*_m_ values (gene flow), i.e. the average number of migrants exchanged among populations per generation, were calculated using Wright's method (1951), where *N*_m_ = (1 − *G*_ST_)/4*G*_ST_. Subsequently, the pair-wise Nei's unbiased genetic distance (Nei 1978) was measured to assess genetic divergence among all studied populations, and a cluster analysis was performed using the unweighted pair-group method with arithmetic average (UPGMA). Bootstrap analysis of the UPGMA tree was performed using MEGA 4 (Tamura *et al*. 2007) with 1000 replicates. The inter-group component of the total variance was qualified by *F*-statistics with the analysis of molecular variance (AMOVA) model (Excoffier *et al*. 2005) using the program GenAlEx v6.4 (Peakall and Smouse 2006), and then the extent of geographical structuring of genetic variation was evaluated. Analysis of molecular variance was calculated within and among the 24 populations studied. The statistical significance of the proportion of variance associated with the fixation index (*F*_ST_) was determined through permutation tests against a null distribution generated by the data.

Principal coordinates analysis from the binary matrix of SRAP was subsequently conducted using GenAlEx v6.4 (Peakall and Smouse 2006). A test of the association between geographic distance and genetic distances assessed the isolation by distance pattern (Slatkin 1993). The null hypothesis of no association was tested with Mantel-like permutations of the genetic (*F*_ST_/(1 − *F*_ST_)) and geographic (ln (geographic distance)) matrices using GENEPOP v4.0 (Raymond and Rousset 1995).

Population structure was analysed using Bayesian clustering analysis as implemented in STRUCTURE v2.2 (Falush *et al*. 2003, 2007; Pritchard *et al.* 2012). The calculation was carried out under an admixture ancestry model and correlated allele frequency model. A burn-in period of 10 000 generations, followed by 50 000 iterations, was used to cluster the population. The assumed number of populations (*K*) was set from 2 to 13. Five independent runs were conducted for each *K* value. Other parameters were set to default values, as suggested by Pritchard *et al.* (2012). The Δ*K* statistic, based on the rate of change of log-likelihood of data [*l*(*K*)] between successive *K* values, was used to select the optimal *K*, following Evanno *et al*. (2005).

The Mantel test using the software ARLEQUIN (Excoffier *et al*. 2005) was adopted to determine the correlation between the Euclidean distances matrix of morphological traits and genetic distance matrix of molecular markers. Finally, a correlation analysis was employed between diversity indexes and environmental factors (such as latitude, longitude, altitude and slope).

## Results

### Morphological analysis

The ANOVA outcomes showed significantly different morphological characters among all studied populations. Highly significant differences occurred at *P*< 0.01 in 32 morphological traits, but not fruit width (FW) or fruit length/width (FLW) (Table 1).

Simple correlation coefficient analysis showed the existence of significant positive or negative correlations among morphological characteristics (data not shown). Sixteen morphological traits (L_H_, LW, L_L_, LSW, LL, β, LA, KFSL, KFW, KFL, LSL, FSL, FL, FW, SL and SW) are important characters that may result in the phenotypic variation of *A. grosseri*.

The individuals in different populations were phenotypically diverse. The CV was the lowest for attachment scar (AS) (CV = 6.84 %), while it was the highest for leaf apex width/height (LAWH) (CV = 37.83 %). The CV of leaf, fruit and seed traits was 21.23, 14.79 and 13.11 %, respectively (Table 1). At the population level, the mean of CV of all populations was 18.19 % (Table 3).

**Table 3.** Locations characters and genetic diversity indexes of all sampled *A. grosseri* populations. *N*, sample numbers; Lat, latitudes; Long, longitudes; Al, altitudes; CV, coefficient of variation; *H*_SW_, Shannon–Wiener index; *N*_a_, observed number of alleles; *N*_e_, effective number of alleles; *H*, Nei's gene diversity; *I*, Shannon information index; PPB, percentage of polymorphic band.

Populations	Locations	*N*	Lat	Long	Al (m)	Aspect	Slope (°)	*CV*	*H*_SW_	*N*_a_	*N*_e_	*H*	*I*	PPB (%)
WH	Weihua, Shanxi	25	112°08′	36°42′	1570	WN	16	14.13	1.71	1.67	1.63	0.34	0.497	67.39
HYDXG	Hongyadaxiagu, Shanxi	25	111°33′	36°29′	1500	NE	15	17.93	1.78	1.76	1.70	0.37	0.52	76.09
JMLC	Jiemiaolinchang, Shanxi	25	111°27′	36°29′	1450	NE	20	15.26	1.74	1.65	1.48	0.41	0.58	65.22
QLY	Qiliyu, Shanxi	28	111°59′	36°37′	1560	WN	10	13.32	1.72	1.68	1.46	0.26	0.38	68.48
SGS	Shigaoshan, Shanxi	30	110°30′	36°15′	1410	N	23	16.49	1.72	1.75	1.73	0.38	0.53	75.00
LKS	Lingkongshan, Shanxi	28	112°05′	36°31′	1450	NE	18	13.47	1.722	1.70	1.49	0.42	0.59	69.57
MS	Mianshan, Shanxi	25	112°01′	36°51′	1380	WN	20	16.77	1.71	1.73	1.50	0.28	0.41	72.83
XC	Xiachuang, Shanxi	30	111°36′	36°16′	1600	NE	25	17.10	1.75	1.79	1.59	0.33	0.47	79.35
CCLC	Cencunlinchang, Shanxi	30	111°28′	36°15′	1170	N	10	17.80	1.71	1.77	1.59	0.33	0.47	77.17
HLSLC	Huanglanshulinchang, Henan	27	122°04′	35°16′	1493	NE	17	19.22	1.76	1.80	1.58	0.32	0.47	80.43
WWS	Wangwoshan, Henan	28	112°26′	35°38′	1346	WN	22	21.72	1.71	1.85	1.65	0.36	0.52	84.78
BYS	Baiyunshan, Henan	25	111°50′	33°40′	1479	N	20	20.12	1.83	1.79	1.63	0.34	0.49	79.35
LJS	Laojunshan, Henan	30	111°38′	33°44′	1352	NE	10	20.53	1.84	1.87	1.66	0.37	0.54	86.96
LJL	Laojieling, Henan	32	111°44′	33°37′	1482	NE	13	18.13	1.84	1.86	1.62	0.35	0.51	85.87
LTG	Longtangou, Henan	30	111°36′	33°31′	1560	WN	25	21.74	1.80	1.89	1.64	0.35	0.52	89.13
TBS	Tongbaishan, Henan	30	113°38′	32°28′	1088	WN	15	21.88	1.84	1.88	1.78	0.41	0.58	88.04
TBD	Taibaiding, Hebei	35	113°37′	33°31′	1023	WN	17	21.46	1.85	1.60	1.43	0.25	0.35	59.78
TTZ	Tiantangzhai, Anhui	30	115°46′	31°08′	1254	N	26	23.92	1.86	1.93	1.87	0.45	0.62	92.39
WCLC	Wochuanlinchang, Anhui	27	115°37′	31°15′	1129	NE	25	21.92	1.84	1.91	1.81	0.42	0.60	91.30
TZS	Tianzhushan, Anhui	30	116°27′	30°44′	1138	WN	21	22.12	1.85	1.90	1.73	0.40	0.57	90.22
TBSH	Taibaishan, Shaanxi	30	117°33′	33°56′	1493	N	15	18.10	1.72	1.80	1.63	0.35	0.50	80.43
HS	Huashan, Shaanxi	25	109°30′	34°29′	1356	WN	17	19.17	1.76	1.82	1.61	0.34	0.48	81.52
LBG	Labagou, Beijing	30	116°36′	40°56′	1468	NE	19	12.65	1.71	1.62	1.44	0.25	0.36	61.96
WLS	Wulingshan, Hebei	20	117°21′	40°32′	1548	NE	22	11.61	1.75	1.64	1.45	0.25	0.37	64.13
Mean								18.19	1.77	1.78	1.62	0.35	0.50	77.81

**Figure 2.**
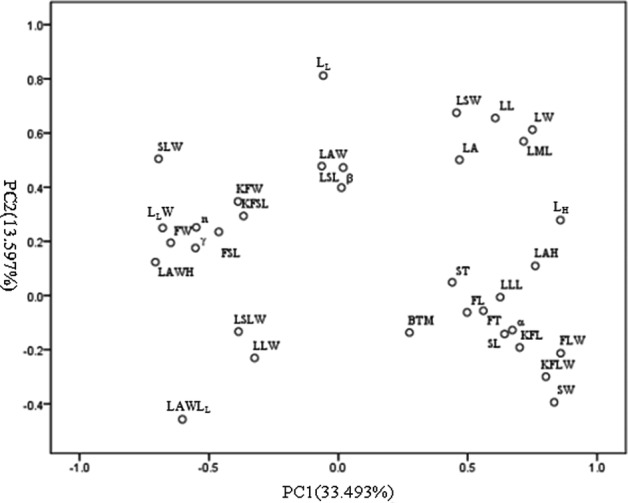
The first two principal component analyses of 34 morphological traits in *A. grosseri*.

For each factor of PCA, a principal component loading of >0.58 was considered as being significant (Barro-Kondombo *et al*. 2010; Jugran *et al*. 2013). In this study, PCA indicated 15 components that explained 85.95 % of the total variance. The first three components explained 60.20 % of the total variation (Table 1, Fig. 2). The first component (PC1) contributed 33.49 % of the variation and involved LL, LML, LW, L_H_, α, LAH, LLL, KFL, KFLW, FLW, SL and SW. The second component (PC2) accounted for 13.60 % of the total variation and featured LL, LML, LW, L_L_ and LSW, while the third component (PC3) accounted for 13.11 % of the variation, featuring LA, γ, AS and FW.

The phenotypic differentiation coefficient (*V*_ST_) of all morphological traits was 36.06 %, indicating that differentiation mainly occurred within populations (Table 1).

All traits were then used to estimate phenotypic diversity (*H*_SW_), and the mean of the Shannon–Wiener index for 34 morphological characters was 6.10, range 4.12–7.13 (Table 1). The Shannon–Wiener index of the 24 populations ranged from 1.71 to 1.86, with a mean value of 1.77 at the population level. The highest phenotypic diversity was found in the TTZ population (*H*_SW_ = 1.86), while the lowest was in the MS population (*H*_SW_ = 1.71) (Table 3).

**Figure 3.**
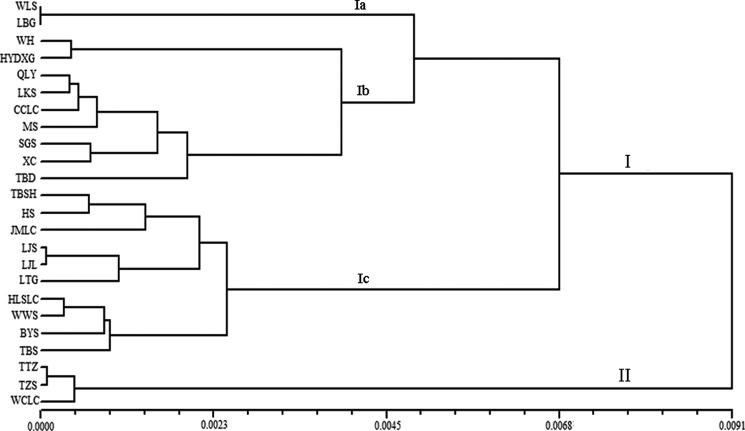
UPGMA-derived dendrogram based on Euclidean distances of the 34 morphological characters of *A. grosseri* populations.

Morphological cluster analysis based on UPGMA showed two distinct clusters among all studied populations (Fig. 3). The first main cluster (I) was divided into three subclusters. Subcluster Ia contained two populations LBG and WLS. Nine populations (WH, HYDXG, QLY, LKS, CCLC, MS, SGS, XC and TBD) were placed in subcluster Ib. Subcluster Ic consists of 10 populations (LJS, LJL, LTG, HLSLC, WWS, BYS, TBS, TBSH, HS and JMLC). The second main cluster (II) contained three populations TTZ, TZS and WCLC.

### Genetic diversity

The SRAP-PCR profile showed high polymorphism in the populations. The primers yielded a total of 140 highly reproducible SRAP bands ranging from 220 to 2000 bp in size. The number of polymorphic bands per primer pair ranged from 8 to 20. The highest genetic diversities were detected by primer pair Me1/Em3, whereas the lowest was detected by Me1/Em11. As summarized in Table 3, 82.14 % (115/140) of the bands were polymorphic (i.e. an average of 5.8 polymorphic bands per primer), indicating a high level of genetic diversity among different populations.

The average gene diversity (*H*_e_) (Nei 1978) was estimated to be 0.35 at the species level. The number of alleles per locus (*N*_a_), effective number of alleles per locus (*N*_e_) and the Shannon's information index (*I*) at the species level were 1.78, 1.61 and 0.50, respectively (Table 3). Within each population, the PPB ranged from 59.78 to 92.39 %, and Shannon's information index (*I*) ranged from 0.35 to 0.62. Nei's gene diversity (*H*) showed similar trends, ranging from 0.25 to 0.45. The observed number of alleles (*N*_a_) ranged from 1.60 to 1.92. The effective number of alleles (*N*_e_) ranged from 1.43 to 1.87. The highest and lowest genetic diversities occurred in populations TTZ (*N*_a_ = 1.60, *N*_e_ = 1.43, *H* = 0.25, *I* = 0.35, PPB = 59.78 %) and TBD (*N*_a_ = 1.92, *N*_e_ = 1.87, *H* = 0.45, *I* = 0.62, PPB = 92.39 %), respectively (Table 3).

### Population structure

Analysis of molecular variance revealed that high genetic differentiation occurred within populations (*P*< 0.001). Of the total genetic diversity, there were 1.74 variance components among populations and 16.08 within populations. Ten percent of the total variation occurred among populations, while 90 % occurred within populations (Table 4). Despite the relatively small variation among populations, the differentiation among populations was still highly significant (*P*< 0.001). When all 24 populations were considered, the coefficient of genetic differentiation among populations (*G*_ST_) was 0.20, representing 19.6 % genetic variability among populations and 80.4 % within populations. Furthermore, the population pair-wise *N*_m_ was 3.42 at the species level.

**Table 4.** Analysis of molecular variance within/among populations of *A. grosseri*.

Source of variance	df	Sum of squared difference	Mean squared difference	Variance component	Percentage of variance	*P*
Among populations	37	780.91	780.91	1.74	10 % (*Φ*_ST_ = 0.10)	<0.001
Within populations	323	3586.53	3586.53	16.08	90 %	<0.001
Total	360	4367.44	4367.44	17.82	100 %	

**Figure 4.**
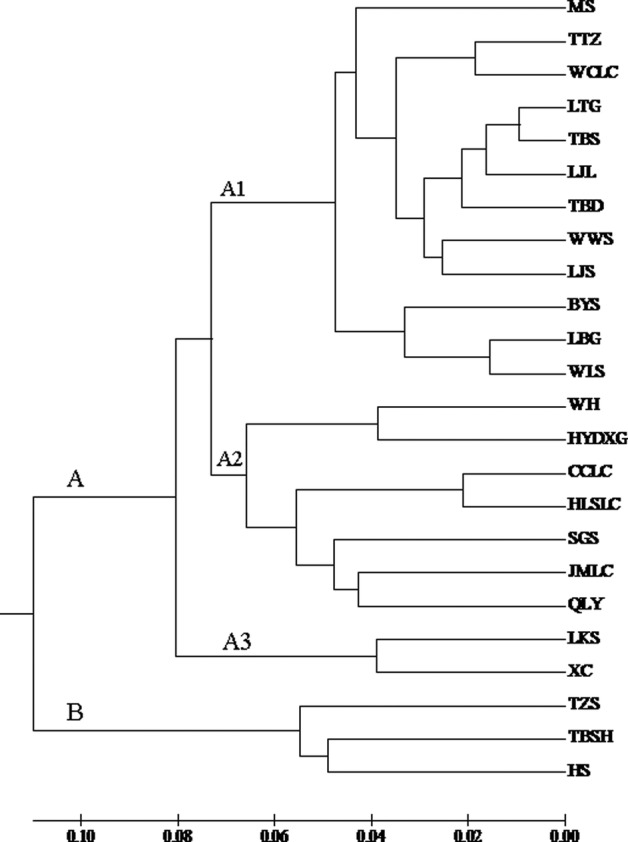
UPGMA dendrogram of *A. grosseri* based on Nei's genetic distance.

To further determine the genetic relationships among the 24 populations, UPGMA clustering was carried out using Nei's unbiased genetic distance matrix. The dendrogram (Fig. 4) clearly shows two groups (A and B). Twenty-one populations were clustered in one group (A). Within Group A, 12 populations (WWS, BYS, LJS, LJL, LTG, TBS, TTZ, WCLC, LBG, WLS, TBD and MS) clustered in a subgroup (A1); 7 (WH, TYDXG, CCLC, JMLC, SGS, QLY and HLSLC) formed another subgroup (A2); LKS and XC formed the third subgroup (A3). Three populations (TBSH, HS and TZS) clustered into another group (B). These findings suggest that individuals in a province are not clustered. Consequently, the Mantel test revealed no significant correlation between pair-wise genetic distances and geographical distances among all populations (*r* = 0.18, *P*> 0.05).

**Figure 5.**
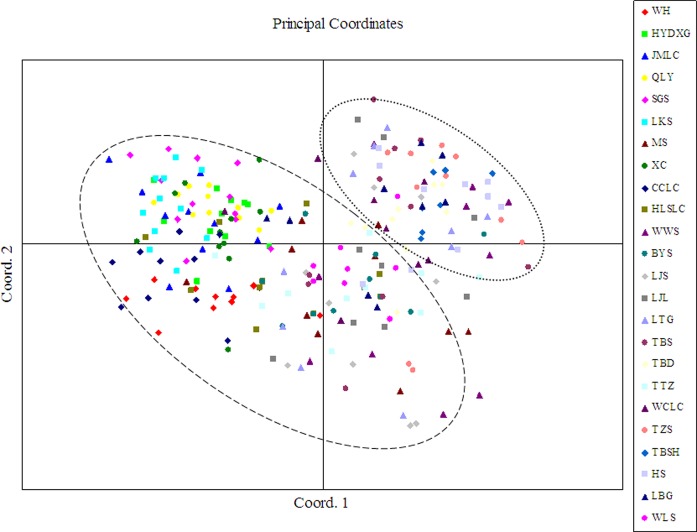
The PCoA for *A. grosseri* individuals. Different colours represent the individuals of different populations.

The genetic relationship among the studied populations was also visualized by performing PCoA based on SRAP data. The first two components accounted for 78.05 % of variation observed in the populations. The two-dimensional plot generated from PCoA also supported the clustering pattern of the UPGMA dendrogram and revealed inter- and intra-population relationships (Fig. 5).

**Figure 6.**
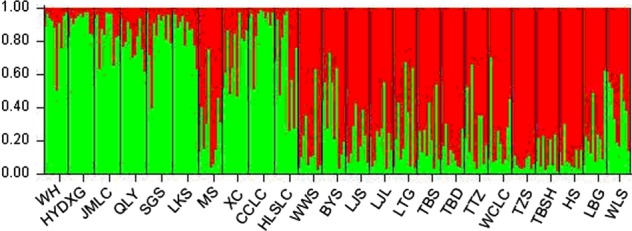
Estimated genetic structure for *K* = 2 obtained with the STRUCTURE program for 24 populations of *A. grosseri*.

For the Bayesian cluster analysis, *K* = 2 was determined as the most likely number of clusters (Fig. 6), according to the *ad hoc* statistic Δ*K* (Evanno *et al*. 2005). This suggested the clustering of the 24 populations into two groups. The topology was basically consistent with the results of PCoA and UPGMA.

Correlation analysis revealed that a significant negative relationship existed among *N*_a_ (*r*= 0.24, *P*< 0.01), *N*_e_ (*r* = 0.51, *P*< 0.01), *H* (*r* = 0.34, *P*< 0.01), *I* (*r* = 0.19, *P*< 0.01), PPB (*r* = 0.48, *P*< 0.01) and longitude, respectively.

There was a significant correlation between the Euclidean distances matrix of morphological traits and the genetic distance matrix of SRAP data according to the Mantel test (*r* = 0.72, *P* = 0.05).

## Discussion

This study offers the first detailed analysis of the genetic diversity and population structure of *A. grosseri* based on both morphological characters and SRAP molecular markers that have been proven to be valuable for the determination of genetic diversity and population differentiation. The comparison of molecular and morphological data for the evaluation of genetic variation has been reported previously (Turchetto *et al*. 2014; Yook *et al*. 2014). In our study, morphological results revealed that obvious variation for most morphological traits and high phenotypic diversity occurred among populations of *A. grosseri* (Tables 1 and 3). The mean phenotypic diversity of *A. grosseri* (*H*_sw_ = 1.77) was higher than that of *A. ginnala* (*H*_sw_ = 1.60) (Wang *et al*. 2012), but lower than that of *A. mono* (*H*_sw_ = 2.68) (Ji *et al*. 2012). High phenotypic diversity within species can enhance adaptation to different surroundings and environmental conditions and increase productivity and ability to colonize new habitats (Stöcklin *et al*. 2009; Ellers *et al*. 2011; Last *et al*. 2014). Based on SRAP markers, 24 *A. grosseri* populations also showed a relatively high level of genetic diversity at the species level. Compared with other species of Acerceae, the genetic diversity of *A. grosseri* was higher than that of *A. ginnala* (Yan *et al*. 2010) and *A. miaotaiense* (Li *et al*. 2005*b*), but lower than that of *Dipteronia dyeriana* (Li *et al*. 2005*a*) and *D. sinensis* (Li *et al*. 2005*b*).

The level and distribution of genetic variability among and within populations can be shaped by a number of factors (Hamrick and Godt 1996) including pollen and seed dispersal, successional stages, geographical distribution range, adult density, mating system, colonization history and natural selection (Hamrick and Godt 1996; Teixeira *et al*. 2014).

The high genetic diversity of *A. grosseri* is probably related to its long evolutionary history, being a long-lived perennial tree that is relatively widely distributed in North China (Fang 1981). Such a long history and wide distribution range may have rendered it possible to accumulate a large amount of genetic variability (Koelling *et al*. 2011; Last *et al*. 2014; Teixeira *et al*. 2014). Despite the shrinking of distribution range due to climate change during the Quaternary period and recent habitat fragmentation and loss, a considerable proportion of the ancestral genetic variability has been well conserved in these *A. grosseri* populations, and thus, high intra-specific genetic diversity was exhibited in this study.

The mating system of a species has a major impact on genetic diversity within the species and populations (Glémin *et al*. 2006; Koelling *et al*. 2011; Last *et al*. 2014). High genetic diversity results mainly from the out-breeding of a species (Glémin *et al*. 2006; Koelling *et al*. 2011). *Acer grosseri* is a dioecious plant. Its flowers are unisexual, with no stamens in the female flowers and no ovary in the male flowers during flowering (Fang 1981). These characters make its mating system favour the out-breeding type.

In addition, the lifespan of *A. grosseri* is long, with overlapping generations. Forest stands generally contain several age classes. Through field investigation, the seedlings, saplings and adults of *A. grosseri* were found to dominate the underlayer of the forest canopy (Tian and Wang 2009). The co-occurrence of different age classes helps meet selective pressures (Ma *et al*. 2008), suitable genes from different individuals would persist and accumulate and thus, the genetic foundation of *A. grosseri* would form and contain high diversity within populations (Ma *et al*. 2008; Turchetto *et al*. 2014).

Based on Nei's gene diversity and AMOVA, genetic differentiation among populations (*G*_ST_) and the fixation index (*Φ*_ST_) signify a genetic structure at this hierarchical level. The results show the patterns of genetic structure, i.e. weak genetic differentiation (*G*_ST_ = 0.20; *Φ*_ST_ = 0.10) (Table 4) occurred among *A. grosseri* populations. Morphological characters also showed less phenotypic variation found among populations (*V*_ST_ = 38.21 %) (Table 1).

In general, genetic differentiation mainly exists within populations of species with a wide geographical distribution (Wright 1978). Growing widely in North China, for example Shanxi, Shaanxi, Hebei, Hubei, Anhui and Henan provinces (Liu 2000), *A. grosseri* showed less genetic differentiation among populations than within populations.

According to Wright (1951), when the gene flow and genetic drift among populations reach equilibrium, a positive correlation between genetic distance and geographical distance should be detected; when such equilibrium is disturbed, however, such correlation is no longer detectable because either gene flow or genetic drift can overwhelmingly influence population structure. In population genetics, a gene flow lower than 1 (*N*_m_ < 1) is generally regarded as the threshold quantity, below which significant population differentiation may occur (Slatkin 1987). In this study, the moderate level of migration among *A. grosseri* populations had occurred, and *N*_m_ was 3.42. *Acer grosseri* undergoes entomophilous pollination and has key fruits (Fang 1981; Liu 2000), indicating that pollen via insects and fruits via wind could disperse over long distances. This may facilitate the connectivity among populations and increase genetic exchange, and eventually contribute to the genetic differentiation within populations. The Mantel test further confirmed the above result, revealing no significant correlation between geographic and genetic distances (*r* = 0.18, *P*> 0.05). However, population decline could occur owing to isolation and artificial destruction of *A. grosseri* populations. Random genetic drift in relatively small populations would result in differentiation between populations. This random factor may be an important reason for the lack of significant correlation between geographic and genetic distances.

Ecological conditions across all sample sites were heterogeneous in terms of temperature, soil reactivity, humidity and light conditions (Ohsawa *et al*. 2007). The selective pressure on populations resulting from a difference in ecological conditions and habitat characteristics thus differed, resulting in differentiation between populations. So, when estimating the genetic diversity of wild plant populations, various environmental factors should be considered (Ohsawa *et al*. 2007). Based on the results of a previous field survey, *A. grosseri* can grow in various habitats and different soil types (Liu 2000; Li *et al.* 2009). Furthermore, the meteorological factors varied among sample sites, for example mean annual air temperature and annual precipitation in LBG were 11.8 °C and 577 mm, respectively, while in TBS, they were 13.4 °C and 573 mm, respectively. We also studied the correlation of genetic structures in plant populations with various environmental factors. The results revealed a significant relation between genetic parameters and longitude. The selective forces from environmental variability may generate the high diversity and genetic pattern in *A. grosseri*.

The clustering pattern derived from morphological and SRAP markers showed 24 populations of *A. grosseri* mainly gathered into two clusters (Figs 3 and 4). Principal components analysis (PCoA) (Fig. 5) and STRUCTURE analysis (Fig. 6) produced groups consistent with the cluster analysis. Our findings demonstrated that morphological characters of *A. grosseri* are reliable sources of information. However, the results of phenotypic and genotypic analyses differed in subclusters. In the case of morphological trait analysis, there was a tendency to group by geographical region, while SRAP marker analysis could not differentiate populations by geographical region. These results suggest that morphological trait analysis alone may not show sufficient genetic diversity in the same species because available traits are limited and some of the traits are not inheritable and vary with different environmental conditions; furthermore, there may be bias in the description of morphological characters (Ravi *et al*. 2003; Baránek *et al*. 2006; Yook *et al*. 2014). Although the phenotypic analysis showed poorer resolution than the genotypic analysis, it is still important because it can be used to analyse genetic diversity and differentiation more quickly and easily.

## Conclusions

Our results indicate that the genetic diversity of *A. grosseri* is high based on morphological characters and genetic data. Less genetic differentiation occurred among the natural populations of *A. grosseri*. All studied populations were mainly gathered into two clusters. The phenotypic variation was obviously positively correlated with the genetic distance based on SRAP markers. High genetic variation within populations could be affected by the biological characters, mating system and lifespan of *A. grosseri*, whereas the lower genetic diversity among populations could be caused by effective gene exchange, selective pressure from environmental heterogeneity and the species' geographical distribution.

## Sources of Funding

This work was supported by the Natural Science Foundation of Shanxi Province, China (2011011031-2) and the undergraduate innovative experiment program of China (201310118004).

## Contributions by the Authors

Q.-d.Z. conducted the fieldwork and analysed data. R.-Z.J. performed the experiments and analysed data. C.M. performed the experiments, analysed data and wrote the manuscript. C.-W.T. joined the fieldwork and performed the experiments. Y.-L.W. conceived, designed the experiments and wrote the manuscript.

## Conflict of Interest Statement

None declared.
